# Lymphatic Trafficking in the Eye: Modulation of Lymphatic Trafficking to Promote Corneal Transplant Survival

**DOI:** 10.3390/cells10071661

**Published:** 2021-07-02

**Authors:** Yanhong Hou, Felix Bock, Deniz Hos, Claus Cursiefen

**Affiliations:** 1 Department of Ophthalmology, Faculty of Medicine and University Hospital Cologne, University of Cologne, 50937 Cologne, Germany; yh_hou@163.com (Y.H.); felix.bock@uk-koeln.de (F.B.); deniz.hos@uk-koeln.de (D.H.); 2 Department of Ophthalmology, Shanghai General Hospital, School of Medicine, Shanghai Jiao Tong University, Shanghai 200080, China; 3 Shanghai Key Laboratory of Ocular Fundus Disease, National Clinical Research Center for Eye Diseases, Shanghai 200080, China; 4 Center for Molecular Medicine Cologne (CMMC), University of Cologne, 50937 Cologne, Germany

**Keywords:** lymphatic trafficking, corneal transplantation, graft rejection, corneal collagen crosslinking, fine needle diathermy, lymphangioregression, preconditioning

## Abstract

(Lymph)angiogenesis into the cornea prior to and after corneal transplantation is a critical risk factor for allograft rejection. Lymphatic vessels even more than blood vessels seem important in mediating immune responses, as they facilitate allograft sensitization in the draining lymph nodes. Thus, the concept of modulating lymphatic trafficking to promote corneal graft survival seems promising. A variety of approaches has been developed to inhibit progressive lymphangiogenesis in experimental settings. Recently, additionally to pharmacological approaches, clinically available techniques such as UVA-based corneal collagen crosslinking and fine needle diathermy were reported to be effective in regressing lymphatic vessels and to experimentally promote graft survival. Clinical pilot studies also suggest the efficacy of blocking antigen presenting cell trafficking to regional lymph nodes by regressing corneal lymphatic vessels to enhance allograft survival in high-risk eyes. In this article, we will give an overview of current strategies to modulate lymphatic trafficking with a special focus on recently reported strategies, which may be easy to translate into clinical practice. This novel concept of temporary, pretransplant regression of lymphatic vessels at the site of transplantation to promote subsequent corneal transplant survival (“lymphangioregressive preconditioning”) may also be applicable to other transplantation sites later.

## 1. Introduction 

The transparent cornea is one of the few avascular tissues of the body. Corneal avascularity, which is also known as the corneal “(lymph)angiogenic privilege”, is crucial for its transparency and thereby visual acuity [1]. In healthy eyes, lymphatic vessels are only observed in the limbus (the transition zone where the conjunctiva and the opaque sclera fade into the clear cornea [1]), the conjunctiva, the extraocular muscles, and the lacrimal gland [2]. The corneal (lymph)angiogenic privilege, which is established early in embryonic development [3], is actively sustained by diverse and redundantly organized mechanisms [1,2] and is attributable to a dynamic balance of pro(lymph)angiogenic and anti-(lymph)angiogenic as well as immunemodulatory factors including vascular endothelial growth factors (VEGF), basic fibroblast growth factor (bFGF), the soluble vascular endothelial growth factor receptors (sVEGFR-1, sVEGFR-2, sVEGFR-3), and pigment epithelium-derived factor (PEDF), within both the cornea and the adjacent aqueous humor [4,5,6,7,8,9]. 

However, the dynamic balance of pro- and anti(lymph)angiogenic factors may be disrupted, e.g., by severe inflammation, which can cause the breakdown of the corneal (lymph)angiogenic privilege and result in pathological corneal lymphatic and blood vessel formation [8,10,11,12]. Several severe inflammatory stimuli, such as infections, inflammation, and chemical burns, can cause the development and ingrowth of lymphatic vessels, which is called lymphangiogenesis. During corneal lymphangiogenesis, an increase in limbal lymphatic extensions occurs towards the central cornea [2,13,14]. Additionally, corneal lymphangiogenesis can be induced by several well-characterized experimental procedures, which can be simply imaged and analyzed because of the transparent nature of the cornea and its easy accessibility [2], which provides the feasibility and benefit for further studies in regards to lymphatic modulation.

Pathological corneal blood vessels are well accepted as a risk factor for corneal transparency loss as well as a cause for secondary transparency-reducing effects including fluid and lipid deposition [1,2]. Moreover, it was reported that the degree of corneal neovascularization prior to corneal transplantation (keratoplasty) correlates with the rejection risk after corneal transplantation [13,14]. Current strategies to control corneal angiogenesis include corticosteroid therapy, anti-VEGF agents (aflibercept, bevacizumab, ranibizumab, pegaptanib, VEGF receptor tyrosine kinase inhibitors) and inhibition of insulin receptor substrate-1 [13]. Additionally, other novel experimental target options, such as netrin-4, soluble CD83, basic fibroblast growth factor (bFGF), and cysteine, also show promising initial results in the suppression of corneal angiogenesis [13,15,16,17,18]. Corneal grafting experiments have been comprehensively conducted to also investigate the role of lymphatic vessels in transplant immunology [19,20,21,22,23,24,25,26,27]. Usually, pathological lymphatic vessels occur in parallel with blood vessels in the cornea, and the extent of lymphangiogenesis seems to correlate with the extent of hemangiogenesis [2]. Unlike blood vessels, which lead to a significant reduction of corneal transparency per se, corneal lymphatic vessels have been shown not to directly affect corneal transparency [28]. However, in addition to (indirectly) mediating graft rejection after corneal transplantation, lymphatic vessels are involved in the pathogenesis of several serious pathological eye conditions, such as dry eye disease [29,30,31,32,33,34,35], ocular allergy [36,37,38], and herpetic keratitis [2,39,40]. Corneal transplantation is the oldest, most commonly performed and overall also most successful form of transplantation. In terms of clinical routine and risk of rejection, there are three major types of surgery performed for corneal transplantation: (1) penetrating keratoplasty (i.e., full-thickness replacement of diseased central cornea); (2) anterior lamellar keratoplasty (i.e., partial-thickness replacement of anterior corneal portion); and (3) endothelial keratoplasty (i.e., partial-thickness replacement of inner corneal layer). Since corneal graft rejections usually target the inner corneal layer (the endothelium), selective replacement of anterior stroma (variant 2) does not carry any risk of immune rejection. Unfortunately, this type of corneal transplantation is performed rarely (about 5%). Worldwide, the most commonly performed type of keratoplasty is replacement of the full thickness of the cornea (variant 1 [41]) Here, the rejection rate strongly correlates with the vascular status prior to transplantation, being low in avascular recipient beds and high in pathologically prevascularized recipient beds. Recently the selective replacement of only the inner corneal layer including the endothelium (version 3) gained much popularity, since transplantation of only this thin layer is also associated with a lower risk of rejection [13]. Again, in pathologically prevascularized recipients, the rejection rates are significantly increased [42,43]. Despite promising outcomes, graft rejection remains the major cause of corneal allograft failure [44]. In addition, the most critical prognostic factor for graft failure is the status of the recipient bed in which the corneal graft is placed [45]. Corneal transplantation into an avascular host bed is generally known as highly successful transplant surgery with allograft survival rates of over 90% after one year and about 80% after 10 years [12]. However, when the corneal graft is transplanted into a pathologically pre-vascularized host bed, graft survival rates are considerably diminished to less than 50% after one year [12], and decrease to 10% in two years or even less [46]. The surgical outcomes of corneal grafts which are transplanted to prevascularized recipient beds are considerably worse than heart, kidney, or liver transplants [45]. Thus, pathological corneal vessels are commonly considered as crucial risk factors that dramatically decrease corneal graft survival [26,27]. 

**Figure 1:**
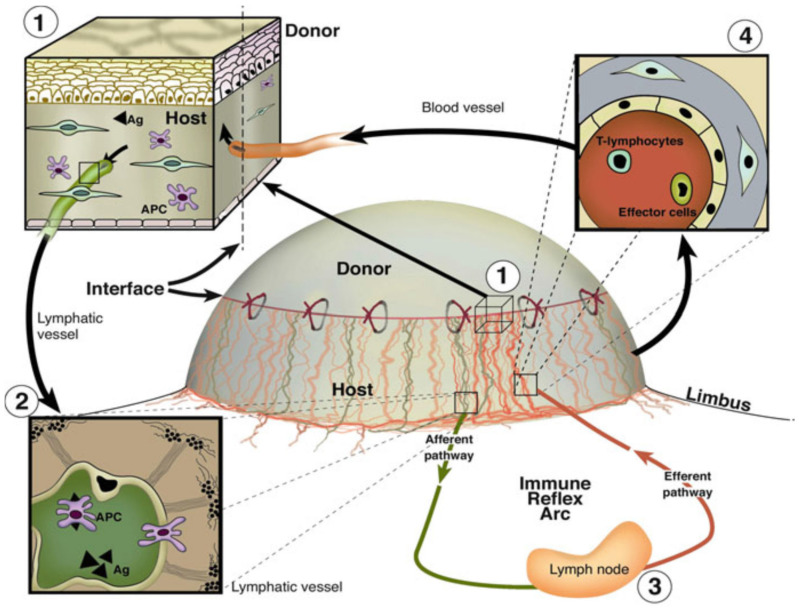
Importance of blood and lymphatic vessels in the host bed of high-risk corneas as exit and entry routes of the immune reflex arc for subsequent immunologic graft rejection. (**1**) Magnification of the host–graft interface: blood (red) and lymphatic vessels (green) reach the host–graft interface. Antigen (Ag) and antigen presenting cells (APCs) both of host and donor can leave the cornea using corneal lymphatics (**2**), conjunctival lymphatics and then as the afferent arc of the immune response reach the regional lymph nodes (**3**). After stimulation of alloreactive T lymphocytes, these and other effector cells/antibodies can reach the graft via corneal blood vessels ((**4**): efferent arc of the immune response). Reproduced from Cursiefen et al. 2003 [48].

Many studies indicate that pathological lymphatic and blood vessels can ease the connection of the corneal graft with the secondary lymphoid organs, resulting in accelerated sensitization against donor antigens and augmented access of immune effector cells to the graft [1,46,47]. Pathological corneal lymphatic vessels, which act as the afferent arm of the immune reflex arc, enable the transport of antigens and antigen presenting cells (APCs) from the donor graft to the host immune system, and therefore facilitate the sensitization process in the draining lymph nodes [26,48,49,50] (Figure 1). Pathological corneal blood vessels, which serve as the efferent arm of the immune reflex arc, facilitate the inflow of inflammatory cells, thereby increasing allospecific immune responses and leading to allograft rejection [48,51]. Thus, pathological corneal lymphatic as well as blood vessels are crucially involved in immune-mediated allograft rejection and dramatically decrease survival rates of corneal transplants [47,51,52].

Importantly, lymphatic vessels have been proven to be even more important than blood vessels in mediating corneal graft rejection [26]. We have previously demonstrated experimentally that corneal grafts transplanted into recipient beds that contain pathological corneal blood vessels but not lymphatic vessels (which was achieved by pharmacological blockade of lymphangiogenesis), resulted in allograft survival rates that were comparable to survival rates in completely avascular recipients. On the other hand, grafts placed into recipient beds with preexisting lymphatic vessels resulted in a significant reduction of corneal graft survival [26]. Consistently, Chen and coworkers could show that blockade of VEGFR-3 signaling significantly inhibited corneal APC trafficking to draining lymph nodes, induction of delayed-type hypersensitivity, and rejection of corneal transplants [53]. In addition to acting as transportation conduits, lymphatic vessels also determine the speed and amount of APCs and other cells or cellular debris reaching the regional lymph nodes in a given time [48]. Since the time window for allograft sensitization after corneal transplantation prior to reestablishment of the ocular immunosuppressive microenvironment is relatively narrow [54,55,56], the early reduction of APC trafficking to draining lymph nodes seems to be important for the risk of graft rejections. In addition, interactions between activated lymphatic endothelium and APCs may further activate them [48,57]. Consequently, selective inhibition or regression of lymphangiogenesis prior to and shortly after transplantation has been shown to promote allograft survival after experimental corneal transplantation [10,27,58,59]. Moreover, in the murine model, removal of the draining lymph nodes also achieved significantly increased survival rates of the corneal allograft, thus further underlining the key role of the lymphatic pathway in mediating allograft rejections [60,61].

In summary, pathological corneal lymphatic vessels facilitate graft sensitization and lead to graft rejection after corneal transplantation. Thus, the concept of modulating lymphatic trafficking of APCs to promote long-term graft survival in high-risk keratoplasty is distinctly promising. Importantly, it is critical to establish effective anti(lymph)angiogenic and (lymph)angioregressive strategies, especially in pre-vascularized high-risk eyes, which may easily be translatable into clinical practice. Here we will review current strategies which modulate corneal lymphangiogenesis and may promote corneal graft survival, so as to better understand lymphatic immune cell trafficking in the eye and specially in the cornea. We discuss the novel concept of promotion of corneal transplant survival by modulating lymphatic trafficking (“lymphangioregressive preconditioning of the host site”). 

## 2. Lymphatic Endothelial and Immune Cell Interaction

APCs like dendritic cells and macrophages are the most important immune cells to induce an allospecific immune response. Similar to lymphatic vessels, the presence of dendritic cells in the cornea was also for a long time unknown. Nowadays, it is known that Langerhans cells and dendritic cells are present in the cornea and can mature due to inflammatory stimuli such as infection or corneal transplantation. Upon activation, dendritic cells increase the expression of the chemokine receptor CCR7 [62]. With this receptor, they start migrating towards the lymphatic vessels along a CCL21 gradient, which is secreted by lymphatic vessels [63]. It was shown that CCR7, CCL21, as well as CCL19 are also expressed in the inflamed cornea [64]. Furthermore, our group could show that the blockade of CCR7 leads to decreased dendritic cell migration to draining lymph nodes and promotes graft survival in low-risk corneal transplantation [58]. 

**Figure 2:**
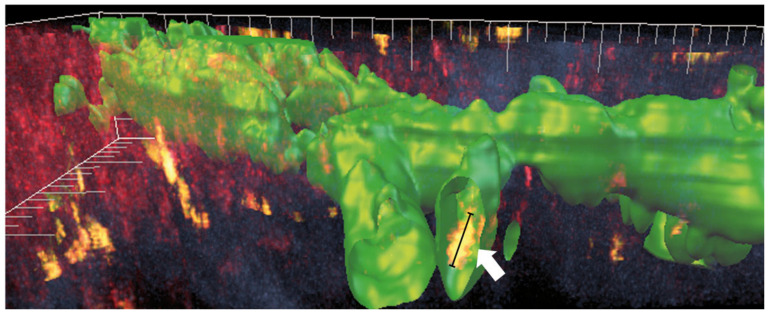
Intravital visualization of lymphatic vessels in the vascularized cornea. By 3D-reconstruction of a 2-photon image stack through the vascularized cornea immune cells (arrow) shown in yellow as an overlay of Alexa488 fluorophore (green) and autofluorescence signal in Alexa488 labeled lymphatic vessels (green), could be observed (Adapted from Steven et al. 2011 [69]).

Blind ending lymphatic capillaries possess loose flaps by which APCs can migrate into the lumen of the lymphatic vessels [65]. Whether this process is active or passive is not fully understood to this day. There are good hints that the Rho-associated protein kinase (ROCK), responsible for actomyosin-mediated nuclear contraction of lymphatic vessels, contributes to transmigration [66]. It was shown that the direct interaction of dendritic cells and lymphatic endothelial cells is mediated by the interaction of leukocyte derived hyaluronan with the lymph-specific marker LYVE-1 [67]. The process of transmigration takes 30–60 min and includes the probing of the lymphatic vessel surface by the dendritic cells prior to entry into the vessel lumen [68]. By intravital two photon microscopy, our group could observe similar behavior in the vascularized cornea. By labeling the corneal lymphatic vessels with an Alexa488 labeled anti-LYVE-1 antibody, corneal lymphatic vessels could be visualized for the first time in vivo. We could show that the immune cells travelled along a labeled lymphatic vessel before entering the lumen via LYVE-1high entry sides. By 3D surface remodeling, the presence of immune cells within the lumen of corneal lymphatic vessels could be demonstrated (Figure 2). Thereby the functionality of pathological corneal lymphatic vessels was proved for the first time [69].

Further important interaction partners for the interaction of dendritic cells and lymphatics are adhesion molecules. It was shown that lymphatic endothelial cells express CD31, CD99, VCAM-1, ICAM-1, L1CAM, and CD137 [70,71,72,73].

Although it is known that VCAM and ICAM are upregulated upon inflammatory stimulation on lymphatic endothelial cells in the skin [71], the expression and role of VCAM and ICAM in corneal lymphatics has not been analyzed so far. Recently, our group could show that the activated leukocyte cell adhesion molecule (ALCAM), binding partner of L1CAM [74,75], is expressed in human corneal LYVE-1+ lymphatic vessels as well as in MHC II+ immune cells. Using a blocking antibody against murine ALCAM, we could show that ALCAM mediates the emigration of dendritic cells via corneal lymphatic vessels. In turn, dendritic cells were trapped in the graft bed, and fewer CD3+ T cells were recruited to the graft bed. Furthermore, inhibiting the interaction between dendritic cells and corneal lymphatics led to the increased generation of regulatory T cells (T regs) and improved graft survival [75]. 

In summary, corneal lymphatic vessels closely interact with immune cells, i.e., dendritic cells. This interaction could become a new target for immune modulatory therapies in and outside the cornea.

## 3. Role of Lymphatic Vessels in Allograft Rejection after Solid Organ Transplantation

Solid organ transplantation usually necessitates the use of systemic immunosuppressive drugs to reduce the risk of allograft rejection. However, this rather unspecific inhibition of immunity might be associated with numerous side effects and a more selective modulation of the immune system; i.e., the specific blockade of allogenic immune responses, is desirable. In this context, recent work in the fields of renal, lung, and cardiac transplantation has demonstrated that lymphangiogenesis after grafting is associated with an increased risk of allograft rejection, implicating that the lymphatic vascular system might be a therapeutic target to improve the outcome of solid organ transplantation. 

In the context of renal transplantation, it has been demonstrated that chronically rejected human renal allografts contain numerous highly proliferating lymphatic vessels [76,77]. These lymphatic vessels also express several chemokines that are important for the guidance of APCs to the draining lymph nodes, such as CCL21. Thus, these studies provided first evidence for increased lymphangiogenesis in renal transplants and suggested a functional role for these lymphatic vessels in APC chemoattraction in transplanted kidneys. 

Dashkevich et al. recently analyzed the association of lymphangiogenesis and allograft rejection after lung transplantation [78]. Importantly, transbronchial biopsies of patients with histologically evident allograft rejection contained a significantly higher density of lymphatic vessels when compared to biopsies without signs of rejection. 

The involvement of lymphangiogenesis in experimental heart transplantation was investigated by Nykänen and coworkers [79]. This study demonstrated that rejection-associated inflammation induced lymphangiogenesis in cardiac allografts. Furthermore, transplantation-associated lymphangiogenesis in cardiac grafts was functional in attracting APCs to the secondary lymphoid organs, followed by the induction of immune responses against the grafts. Importantly, inhibition of lymphangiogenesis by overexpression of sVEGFR-3 significantly prolonged cardiac allograft survival in rats by reduced APC trafficking to the secondary lymphoid organs. 

Taken together, APC trafficking via lymphatic vessels to the secondary lymphoid organs seems to be an important step for the induction of alloimmune responses, e.g., after renal, lung, or cardiac transplantation. Thus, interference with this pathway, i.e., via targeting lymphangiogenesis, might be a potential strategy to reduce the risk of allograft rejection also after solid organ transplantation. However, it should be noted that in contrast to the cornea, solid organs are (lymph)vascularized in their healthy state and rely on proper lymphatic function and drainage, which has also been shown in the respective transplant settings. In this context, work from Stuht and colleagues demonstrated that human kidney grafts with high lymphatic vessel density have better functional outcomes than those with low lymphatic vessel density 1 year post transplantation [80]. Similarly, work from Geissler et al. indicated that human cardiac allografts with higher lymphatic vessel densities 0.5 months after grafting are less susceptible to rejection episodes during the first postoperative year [81]. It has also recently been reported in a murine orthotopic lung transplantation model, that a decline in lymphatic vessels is associated with increased graft failure rates [82]. Moreover, stimulation of lymphangiogenesis was even able to attenuate allograft rejection in this model. 

Taken together, the role of the lymphatic vascular system in solid organ transplantation is less clear when compared to corneal transplantation and still under investigation. It seems that a complete and permanent blockade of lymphangiogenesis and lymphatic function in solid organ transplantation to improve graft survival might not be applicable [83]. Instead, a more specific interference with the lymphatic vascular system and trafficking of APCs through lymphatic vessels, e.g., in a more time-restricted manner, may be more feasible [83]. 

## 4. Targeting Active Corneal (Lymph)angiogenesis to Promote Corneal Transplant Survival—Concept and Current State of Art

In a simplified scheme, the three structural elements of the immune system that enable immune responses against alloantigens after transplantation are afferent lymphatic vessels, regional lymph nodes, and efferent blood vessels [26,48]. As described above, corneal (lymph)angiogenesis and pathological corneal lymphatics facilitate immune cell trafficking and thereby critically increase the incidence of allograft rejection in high-risk keratoplasty. Therefore, the concept of targeting (lymph)angiogenesis for pre-and post-transplant treatment seems to be promising for the promotion of graft tolerance. 

Since we and others could show that (1) pathologic corneal lymphatic vessels act as conduits for APC trafficking from donor tissue to host regional lymph nodes, (2) that inhibition of lymphangiogenesis prior to and after transplantation leads to improved graft survival, and (3) that a temporary blockade or regression of corneal lymphatic vessels is sufficient to prevent corneal allosensitization, we developed the novel therapeutic concept of “temporary preoperative lymphangioregression at the recipient site to promote subsequent corneal transplant survival” [9,21,22,23,24,25,59,83,84,85,86,87,88,89,90,91,92,93]. This concept of “lymphangioregressive preconditioning” may also be useful for other transplant sites outside the eye [2,83].

A variety of therapeutic strategies for (lymph)angiogenesis inhibition have been developed and are currently being tested to improve graft survival by blocking immune cell trafficking in pathologic corneal lymphatic vessels by either (i) inhibiting progressively outgrowing immature corneal vessels, and/or by (ii) induction of vascular regression of already existing mature pathologic corneal vessels.

Various approaches have already been tested to suppress progressive corneal immature lymphatic and blood vessels. Anti-inflammatory options as well as anti VEGF-strategies have already been tested experimentally and partially also used in the clinic already [10,94,95,96]. Moreover, several anti(lymph)angiogenic agents were preclinically tested, such as soluble VEGFRs, anti-VEGFR3 antibodies, VEGFR-tyrosine kinase inhibitors, and integrin blocking peptides [10,13,20,84,89,97]. 

**Figure 3:**
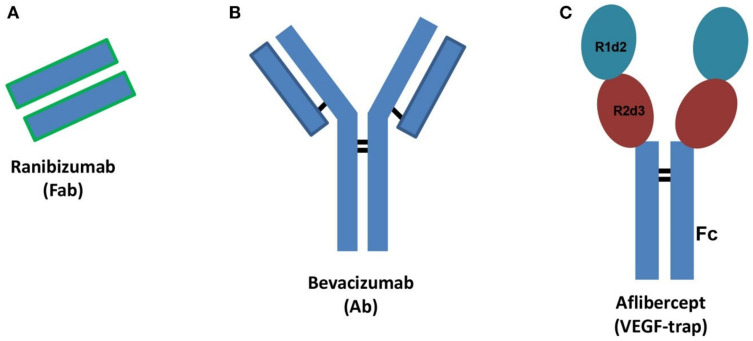
Schematic structures of ranibizumab (**A**), bevacizumab (**B**), and aflibercept (**C**). Ab: antibody, Fab: fragment antigen binding, Fc: fragment crystallizable region. R1d2: domain 2 of vascular endothelial growth factor receptor VEGFR1, R2d3: domain 3 of VEGFR2. Black bars: interchain disulfide bridges. From Platania et al. 2015 [98].

VEGF Trap(R1R2) was reported to be effective in inhibiting corneal lymphangiogenesis via neutralizing VEGF-A in a murine model and was also shown to promote corneal graft survival [27,59]. To date, the only VEGF Trap(R1R2) approved for ophthalmic clinical practice is aflibercept, which is a fusion protein known as VEGF-trap. It works as a decoy VEGF receptor where two binding domains, the domain 2 (d2) of VEGFR1 and the domain 3 (d3) of VEGFR2 (from N-terminus to C-terminus of primary sequence), are connected to the fragment crystallizable region (Fc) of human immunoglobulin (Ig) (Figure 3) [98,99]. In addition, our group has recently demonstrated that locally restricted VEGF depletion via preoperative VEGF Trap(R1R2) application improves corneal transplantation success by modulating the corneal microenvironment in the recipient and inducing tolerogenic mechanisms [23]. It has been reported that application of sVEGFR2 as well as sVEGFR3 resulted in a significant decrease of corneal lymphangiogenesis and enhancement of corneal graft survival [10,100]. Direct blocking of VEGF-C by using anti-VEGF-C (VGX-100) also effectively decreased lymphangiogenesis and enhanced graft tolerance [101]. In addition to their antilymphangiogenic effect, anti-VEGF-C approaches have also been shown to inhibit trafficking and maturation of APCs [102]. Bevacizumab and ranibizumab, which are directed against VEGF-A, were recently tested in clinical settings and demonstrated a comparable potent inhibition of both corneal hem- and lymphangiogenesis [89,103]. It is important to note that bevacizumab is a humanized, recombinant, monoclonal antibody against VEGF-A, which has been approved by the U.S. Food and Drug Administration [103], while ranibizumab is the mutated Fab (fragment antigen-binding) of the recombinant, monoclonal anti-VEGF antibody (Ab) originating bevacizumab (Figure 3) [89,98]. Subconjunctival injection of bevacizumab significantly decreases the corneal neovascularized area and improves graft survival [104], which has been recently translated into the clinic [96,105] and is frequently applied off label to treat patients with corneal neovascular diseases also in the context of keratoplasty [2,28]. It is worth noting that approved anti-VEGF agents used in clinical practice, such as ranibizumab, bevacizumab, and aflibercept, are considerably different in terms of molecular interactions when they bind with VEGF [98]. Therefore, characterization of such features can improve the design of novel biological drugs potentially useful in lymphangiogenesis. Platania et al. analyzed the interaction of the main mediator of angiogenesis—VEGF-A—with binding domains of anti-angiogenic agents, such as ranibizumab, bevacizumab, and aflibercept, which are used for treatment of retinal diseases [98]. They reported that anti-angiogenic agents were considerably different both in terms of stabilizing energy and molecular interactions, which suggests detailed understanding of such drug–target interactions may help in developing novel biological drugs [98]. Additionally, thrombospondin-1 (TSP-1) was shown to be efficient in inhibiting hemangiogenesis in the experimental setting [106]. TSP-1 also blocks lymphangiogenesis as an endogenous inhibitor, which can ligate CD36 on monocytic cells [106], thereby suppressing corneal lymphangiogenesis. There are also studies reporting that via the modulation of integrin α5β1 [107,108], integrin α9 [109], semaphorin 3f (Sema3F) [110], podoplanin [111], matrix metalloproteinases (MMP-2 and MMP-9) [112], and miRNAs [2,13], corneal lymphatic vessel growth can be inhibited and thereby corneal transplant survival promoted. Despite the massive experimental progress, none of these agents has yet been approved by the Food and Drug Administration (FDA) or European Medicines Agency (EMA) for clinical application in the cornea [113,114]. 

In the clinic, topical application of corticosteroid eye drops is so far still the standard antiangiogenic therapy of the cornea. Our group also showed in experimental settings that these drugs are potent inhibitors of corneal lymphangiogenesis, because of direct inhibition of the lymphatic endothelium, as well as decreased recruitment of pro-lymphangiogenic inflammatory cells [90]. Nevertheless, long-term use of corticosteroids may cause side effects such as cataract and increased intraocular pressure. Blockade of insulin receptor substrate-1 (IRS-1) signaling by GS-101 (aganirsen), an anti-IRS-1 antisense oligonucleotide, inhibits angiogenesis and lymphangiogenesis in animal experiments [88,115]. Aganirsen has already been successfully analyzed in in phase II/III trials, which showed safety and efficacy in inhibiting progressive corneal angiogenesis [87,116]. 

Though the above reviewed approaches are able to stop progressive corneal (lymph)angiogenesis and may enhance graft survival after keratoplasty, it is still challenging to regress already existing mature corneal lymphatic and blood vessels. Therefore, our group has recently investigated the effect of several already clinically available techniques on the regression of corneal lymphatic and blood vessels, such as ultraviolet-A (UV-A) light based corneal collagen crosslinking (CXL), fine needle diathermy (FND), and photodynamic therapy (PDT). In the next sections, we will summarize the promising (lymph)angioregressive effects of CXL and FND and their significant improvement of corneal graft survival after pre-transplant treatment, both in the experimental setting (Section 5) and in clinical pilot trials (Section 6). 

## 5. Experimental Modulation of Lymphatic Trafficking via UVA-Light Crosslinking (CXL) and Fine-Needle Diathermy (FND) to Regress Pathologic Corneal Lymphatics to Promote Corneal Transplant Survival: The Eye as a Model of Organ Transplant Immunology (“Lymphangioregressive Preconditioning”)

As aforementioned, lymphatics critically participate in the mediation of allogenic immune responses, which can lead to corneal transplant rejection and failure. Therefore, modulation of lymphatic trafficking is a new and feasible strategy to promote graft survival. Currently, CXL and FND are promising approaches to achieve significant improvement of corneal transplant survival via modulation of lymphatic trafficking in the context of corneal transplantation. 

### 5.1. Preoperative UV-A-Light Based CXL Regresses Corneal Lymphatic Vessels in Prevascularized High-Risk Eyes and Significantly Improves Subsequent Graft Survival in Experimental Settings

**Figure 4:**
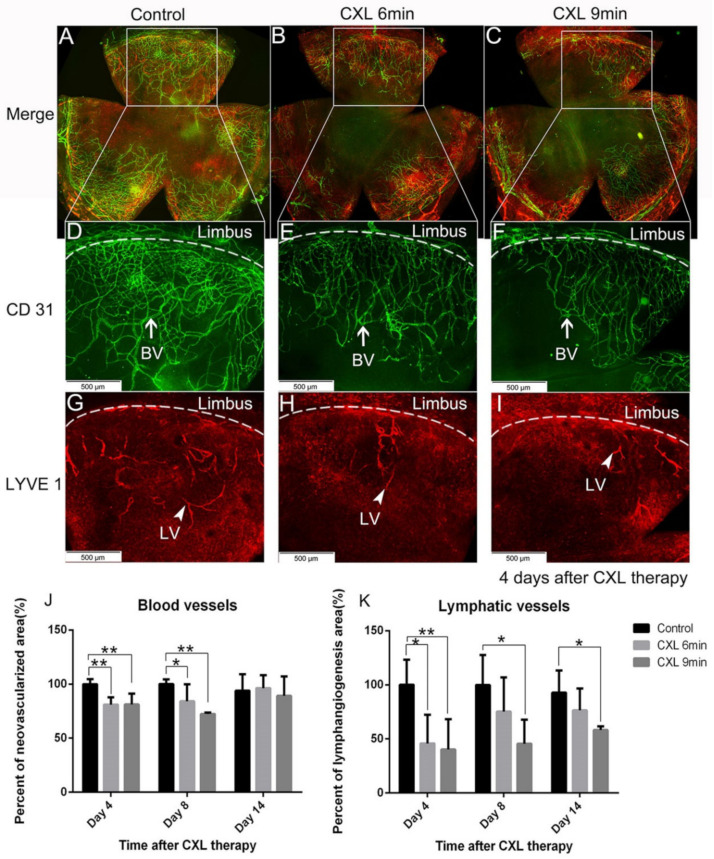
Regression of mature corneal blood (BV) and even more so lymphatic vessels (LV) after UV-A-light based corneal collagen crosslinking (CXL). Corneal whole mount staining after induction of inflammatory corneal neovascularization was performed to quantify both corneal blood and lymphatic vessels via immunohistochemistry. Corneal vessels were double stained 4 days after CXL treatment ((**A**–**C**): merged images). Blood vessels were stained with FITC-conjugated CD31 ((**D**–**F**): BV with white arrow in green) and lymphatic vessels were stained with lymphatic vessel endothelial hyaluronan receptor 1 (LYVE-1) followed by Cy3-conjugated secondary antibody ((**G**–**I**): LV with white arrowhead in red) (magnification: ×100; scale bar: 500 μm). Corneal lymphatic vessels in the 6-min CXL-treated group showed a significant reduction on day 4 post CXL but not on day 8, while in the 9-min CXL-treated group, lymphatic vessels were regressed significantly on both day 4 and day 8 after treatment (**K**) (*n* = 5; * *p* < 0.05, ** *p* < 0.01). At 14 days after CXL treatment, corneal lymphatic vessels in the 9-min CXL-treated group were still significantly reduced compared with the control group, while there was no significant difference in the 6-min CXL-treated group (**K**). Mature corneal blood vessels were regressed significantly on day 4 and day 8 after a 6-min or 9-min CXL treatment (**J**). (*n* = 5; * *p* < 0.05). Reproduced from Hou et al. 2018 [24].

UV-A-light based CXL is often applied in the clinic to stabilize the corneal structure via bonding of adjacent corneal collagen fibers for corneal ectatic diseases through the release of reactive oxygen radicals [117]. Since 2003, CXL has been used as clinical treatment for progressive keratoconus [118]. Topical application of the photosensitizer riboflavin followed by UV-A irradiation is now commonly used as the standard CXL procedure in the clinic for keratoconus. Riboflavin has been approved by the FDA since 2016. Interestingly, CXL has been reported to decrease the density of corneal keratocytes by inducing apoptosis [118,119,120,121]. As corneal blood and lymphatic vessels are also located in the same layer as keratocytes—namely in the corneal stroma—we speculated whether it is possible that these vessels may also be regressed by similar mechanisms, namely via inducing apoptosis of vascular endothelial cells. Indeed, CXL was recently reported by our group to be effective in regressing corneal lymphatic and blood vessels in mice [24] (Figure 4). Using topical riboflavin application followed by UV-A illumination (370 nm, 3 mW/cm^2^, irradiated for 6 or 9 min), pathological corneal lymphatic as well as blood vessels were significantly regressed after CXL in the mouse model of suture-induced inflammatory corneal neovascularization. 

**Figure 5:**
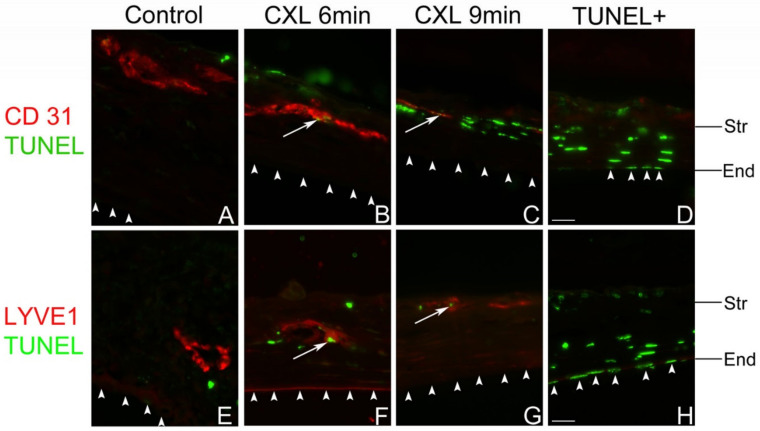
Apoptosis of vascular endothelial cells in corneas after UV-A-light based CXL. Representative terminal deoxynucleotidyl transferase dUTP nick end labeling (TUNEL) assay images of the central corneas from murine corneas 1 day after UV-A-light based CXL for 6-min (**B**,**F**) or 9-min (**C**,**G**) and non-crosslinked control corneas (**A**,**E**). White arrows indicate TUNEL (green) and CD31+ (**A**–**D**) or lymphatic vessel endothelial hyaluronan receptor 1 (LYVE1) + (**E**–**H**) (red) vascular endothelial cells in each panel. Corneal nonvascular endothelial cells are TUNEL negative in all groups; (**D**,**H**) positive control of TUNEL staining (Str: stroma; End: nonvascular endothelium. Colocalization: white arrow. Corneal nonvascular endothelium: white arrowhead. Magnification: ×600. Scale bar: 20 μm). Corneal epithelium is missing in the treatment groups due to corneal abrasion. From Hou et al. 2018 [24].

TUNEL positive signals colocalized with both corneal lymphatic and blood vascular endothelial cells, which indicates that the (lymph)angioregressive effect of CXL is caused by cell death of vascular endothelial cells [24] (Figure 5). CXL also reduced the numbers of macrophages and other CD45 positive leukocytes in pre-vascularized corneas [24] (Figure 5). The reduction of macrophages may also contribute to the regression of pathological corneal lymphatic vessels, as macrophages crucially participate in the formation and maintenance of inflammatory lymphatic vessels [122,123]. 

**Figure 6:**
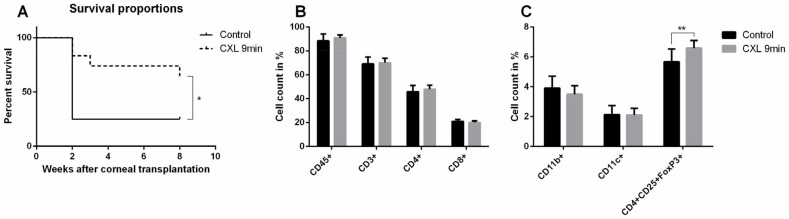
Promotion of graft survival and upregulation of T regulatory cells in vascularized high-risk eyes after pretreatment with UV-A-light based corneal collagen crosslinking (CXL; lymphangioregressive preconditioning). (**A**) The 9-min CXL treatment before corneal transplant significantly promoted corneal allograft survival in pretreated high-risk eyes 8 weeks after corneal transplantation (control: 25.0% graft survival, 9-min CXL: 66.7% graft survival; *n* = 12; * *p* < 0.05). (**B**,**C**) There were significantly more CD4+CD25+FoxP3+ cells in draining lymph nodes in the 9-min CXL-treated group than in the control group at 8 weeks post corneal transplant (*n* = 12; ** *p* < 0.01). FACS analysis showed no statistical difference for CD3+, CD4+, CD8+, CD11b+, CD11c+, or CD45+ cells. From Hou et al. 2018 [24].

Importantly, we observed a significant enhancement of long-term allograft survival using CXL to treat pre-vascularized recipient beds preoperatively [24] (Figure 6). This seems to be due to the regression of pathological corneal lymphatic and blood vessels and the reduction of inflammatory cells in corneal recipient beds [24]. Furthermore, after allogenic corneal transplantation, Tregs in draining cervical lymph nodes were more frequently observed in CXL treated mice compared to controls. This might also have contributed to the promotion of long-term allograft survival in high-risk corneal transplantation, as Tregs can suppress the efferent arm of immune response [124,125,126,127,128,129,130].

Therefore, temporary preoperative (lymph)angioregression via CXL prior to high-risk corneal transplantation to facilitate corneal graft survival shows the feasibility of this preconditioning approach. As the aforementioned beneficial effects of pre-surgically applied CXL on subsequent corneal transplantation have only be shown in animal studies so far, clinical studies are needed to prove the efficacy of this promising approach. In Section 6, we will present the preliminary clinical data of CXL usage in the transplant context [91]. 

### 5.2. FND in Combination with Anti-VEGF Antibody (Bevacizumab) Application Improves Corneal Graft Survival via Lymphatic Trafficking Modulation in Mice 

**Figure 7:**
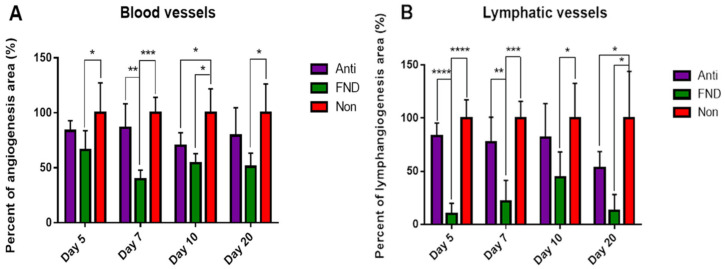
Effect of fine needle diathermy (FND) on regression of blood vessels (**A**) and lymphatic vessels (**B**) at four time points after treatment (after 14 days of corneal suture placement to induce inflammatory corneal neovascularization). Compared to the non-treated group (NON: red column), the FND treated group (green column) resulted in significant regression of both blood and lymphatic vessels at all four time points (*p* < 0.05; Anti: anti-inflammatory treatment only, purple column). The most obvious effect of FND is observable at day 7 with the reduction of hemangiogenesis by 60% (*p* < 0.0001) and lymphangiogenesis by approximately 80% (*p* < 0.0001) as compared to the NON group. (* *p* < 0.05; ** *p* < 0.01; *** *p* < 0.001; **** *p* < 0.0001). From Le et al. 2018 [25].

FND was described and clinically conducted to treat corneal blood vessels firstly in 2000 [131]. It is considered as an inexpensive and simple clinical therapy. As FND is feasible to reach any depth of the cornea, it can occlude any corneal vessel at any corneal depth and is effective in reducing both progressive as well as pre-existing, mature corneal vessels [132]. FND is applied by using a diathermy current and stainless steel needle to coagulate corneal vessels [13]. In addition, our group demonstrated recently that FND can not only destroy visible corneal blood vessels, but also clinically invisible lymphatic vessels in the mouse model of suture-induced inflammatory corneal neovascularization [25] (Figure 7). Here, FND was performed with the needle inserted intrastromally near and in parallel to the limbus. The contact was maintained until mild blanching of the corneal stroma and/or corneal shrinkage occurred [25]. This procedure was repeated until 360-degree circumferential cauterization was reached. In this study, the optimal regressive effect on corneal lymphatic and blood vessels was observed seven days post FND treatment [25]. It is known that after FND, retreatment might be needed, as reperfusion of previously occluded vessels may occur. It has been reported that after one single FND treatment, the success rates of vessel occlusion varies from 60% to 100% [131,132,133]. When applied alone, FND may stimulate the release of proangiogenic factors, especially when used extensively to treat many vessels [131]. Therefore, it is suggested to use corneal angiography to guide FND to selectively target the feeder vessels, which might help in minimizing the quantity of intrastromal needle insertions and diathermy without influencing the efficacy of FND [134]. The combination of FND with topical or subconjunctival bevacizumab is also proposed to antagonize the reactive upregulation of VEGFs, which was described to occur after FND [28,135,136]. 

**Figure 8:**
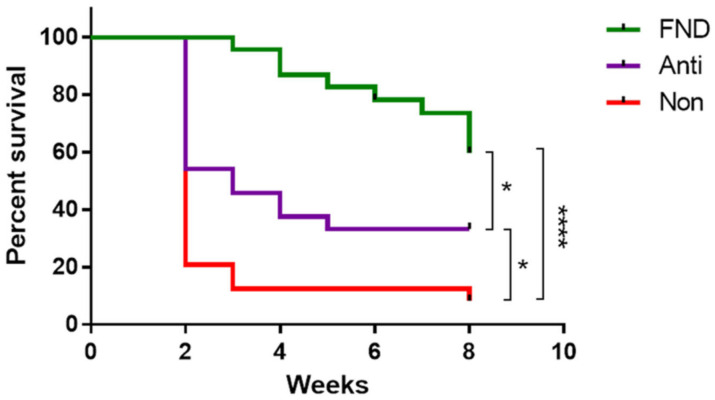
Preoperative fine needle diathermy of pathologic corneal blood and lymphatic vessels in vascularized high-risk eyes promotes graft survival after subsequent allogenic high-risk corneal transplantation. Two weeks after corneal suture placement to induce inflammatory corneal neovascularization, mice were treated with fine needle diathermy and anti-inflammatory drops (FND; green line) or with anti-inflammatory eye drops only (ANTI; purple line). Graft survival was compared to a control group which did not receive FND or anti-inflammatory therapy (NON; red line). Graft survival was significantly prolonged when transplants were placed into recipient beds of pretreated groups (FND versus NON: 60.9% vs. 8.3%, *p* < 0.0001; ANTI versus NON: 33.3% vs. 8.3%, *p* = 0.0164). Between the two treated groups, our results showed that FND treatment significantly improved the graft survival in comparison to isolated anti-inflammatory therapy (FND versus ANTI: 60.9% vs. 33.3%, *p* < 0.05). (* *p* < 0.05; **** *p* < 0.0001). From Le et al. 2018 [25].

Importantly, we have recently demonstrated that preoperative FND treatment of pathologically pre-vascularized high-risk recipients can significantly enhance allograft survival after subsequent corneal transplantation in mice [25] (Figure 8). Further studies may be helpful to show whether additional postoperative therapy can further enhance graft survival. Beyond that, as FND is already clinically used in patients to treat corneal neovascularization, translational clinical studies analyzing its effect on graft survival should be feasible. In fact, a clinical pilot study by our group has demonstrated promising initial results (see Section 6.2) [47].

Taken together, these studies show that the concept of modulating lymphatic trafficking via CXL and FND prior to high-risk keratoplasty to improve corneal transplant survival is feasible and promising. Translational clinical trials should follow to further prove the effectiveness of this approach.

## 6. Translation of Temporary Pretransplant (Lymph)angioregressive Therapy at the Recipient Site via UVA-Based CXL and FND Approaches into Clinical Practice 

### 6.1. Clinical Pilot Study of Peripheral UV-A-Light Based CXL Shows the Possibility of Enhancing Transplant Survival in High-Risk Eyes

**Figure 9:**
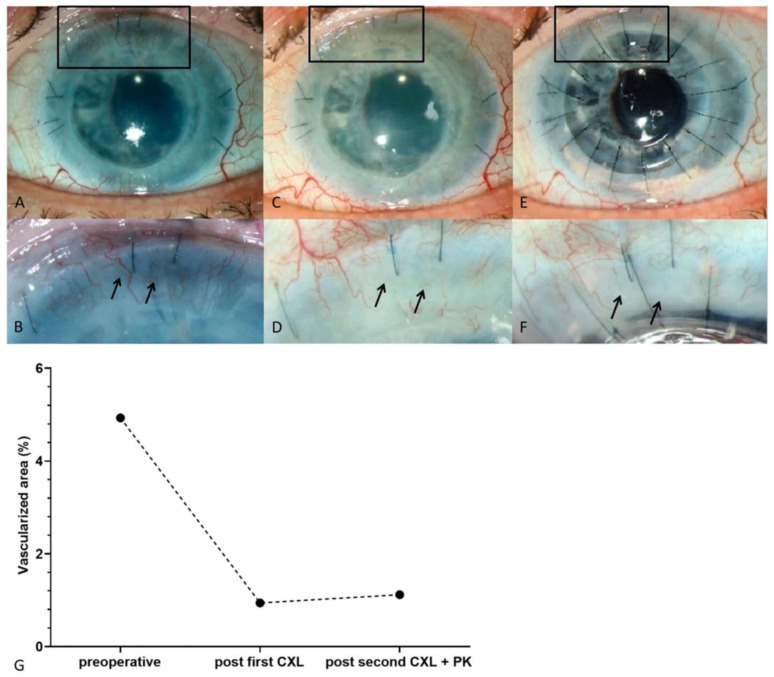
Angioregressive effect of isolated peripheral UVA-light based CXL in a high-risk cornea after 7 failed previous grafts. (**A**) vascularized cornea with marked corneal edema after failure of previous graft. (**B**) Magnification of insert in (**A**); arrows depict a stromal corneal vessel. (**C**) Three weeks after peripheral CXL excluding the limbal vascular arcade; significant regression of corneal neovascularization is observed. (**D**) Magnification of insert in (**C**); note that the blood vessel from (**B**) has disappeared (arrows). (**E**) Two weeks after second peripheral CXL combined with re-keratoplasty. Corneal blood vessels are still absent without revascularization, and the transplant is clear. (**F**) Magnification of insert in (**E**); the blood vessel is still absent (arrows). (**G**) Morphometric quantification of corneal blood vessels shows marked reduction of corneal neovascularization. From Schaub et al. 2018 [91].

**Figure 10:**
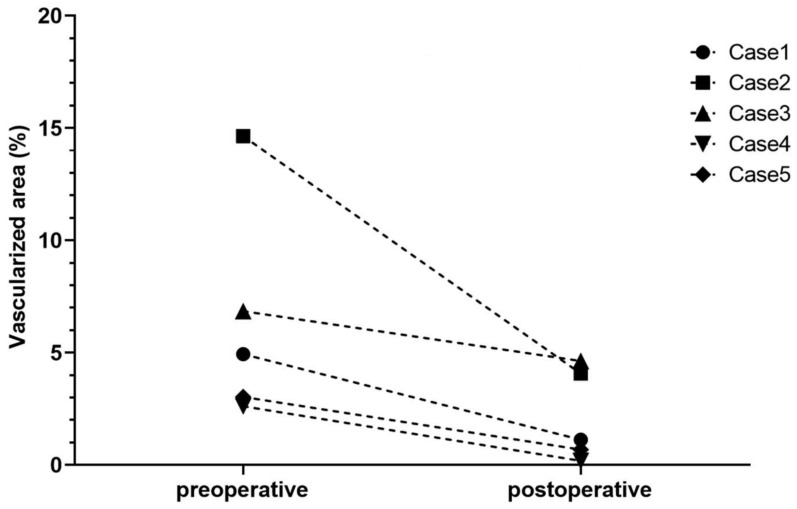
Reduction of corneal neovascularization by peripheral CXL before high-risk PK. In case 1, 1 peripheral CXL was first performed as a single procedure, followed by an additional peripheral CXL procedure combined with keratoplasty. In cases 2 to 5, the peripheral CXL procedure was directly combined with keratoplasty (without previous peripheral CXL). Corneal neovascularization was morphometrically measured as the corneal area covered by blood vessels in relation to the total corneal area. Morphometrical analysis revealed a neovascularization reduction of 70.5 ± 22.7%. From Schaub et al. 2018 [91].

As aforementioned, CXL using UV-A-light and riboflavin as photosensitizer was demonstrated as an effective preoperative strategy to regress pathological corneal lymphatic and blood vessels and promote graft survival in high-risk keratoplasty in the experimental setting [24]. Our group recently reported the first successful clinical case series of neovascularization regression prior to or combined with keratoplasty in pre-vascularized high-risk eyes by peripheral CXL [91]. This retrospective case series included five patients with progressive corneal neovascularization and the need for high-risk corneal transplantation because of previous graft rejection and/or keratitis [91]. Peripheral CXL was applied before or in combination with penetrating keratoplasty. A significant regression of corneal blood vessels was observed after peripheral CXL treatment (Figure 9 and Figure 10). Moreover, this regressive effect of blood vessels was maintained up to several months without revascularization. No side effects were observed during the study duration, and no notable intra- or postoperative complications occurred. Furthermore, all corneal transplants remained clear without any signs of immune reactions (although follow-up after transplantation was rather short in this study). Thus, this study demonstrates that peripheral CXL was highly effective in reducing pathological corneal neovascularization and is safe and well-tolerated, even in eyes with thin and unstable corneas. 

Though this study surely has its limitations, such as small case number and heterogeneity of treated patients in terms of preexisting keratitis, underlying disease, previous surgeries, and timing of keratoplasty and CXL, this pilot study presents the first clinical evidence that CXL is an effective method to reduce pathological corneal neovascularization, to stabilize the recipient cornea, and presumably to promote graft survival after subsequent high-risk keratoplasty. These promising results warrant large-scale clinical studies with longer follow-up. 

### 6.2. Interventional Clinical Study of FND Combined with Bevacizumab Indicates Increase of Rejection-Free Corneal Graft Survival Rates in Lymphangioregressively Pretreated Recipient Eyes

**Figure 11:**
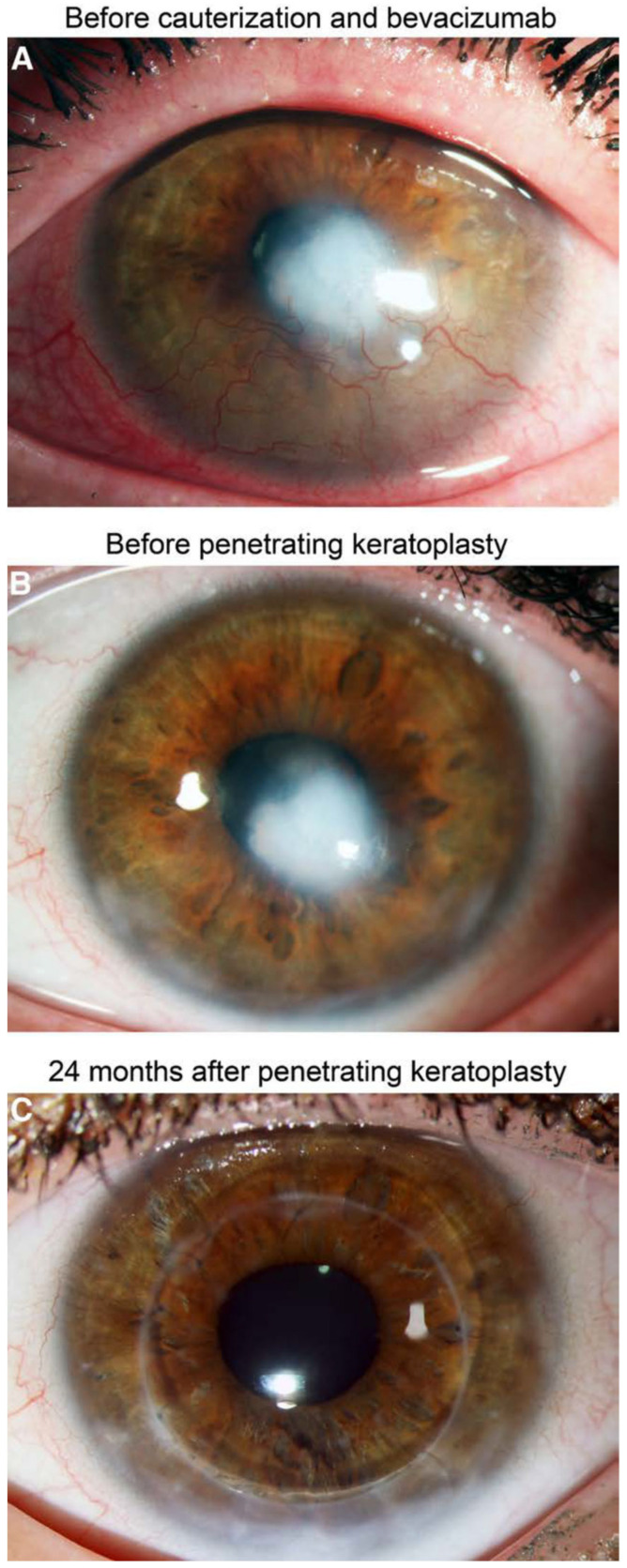
Clinical follow-up of a patient with corneal neovascularization treated with fine-needle diathermy (FND) and bevacizumab application before penetrating keratoplasty. In this eye, no graft rejection was observed. (**A**) Herpetic corneal scar with corneal neovascularization in the lower corneal quadrants before FND and bevacizumab application. (**B**) Corneal scar without neovascularization 6 months after FND and bevacizumab application (1 day before penetrating keratoplasty). (**C**) Clear cornea without any signs of allograft rejection 24 months after penetrating keratoplasty. From Hos et al. 2019 [47].

**Figure 12:**
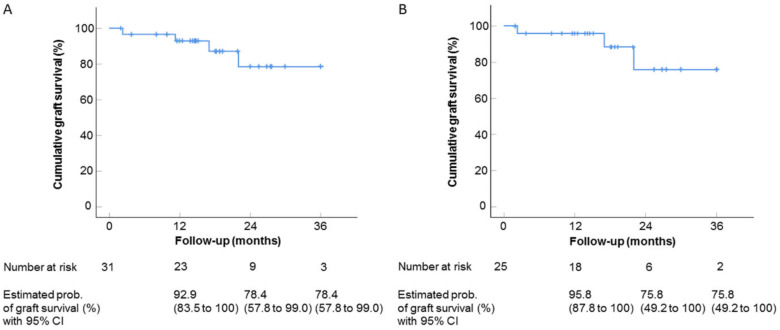
Impact of pre-transplant fine-needle diathermy (FND) combined with subconjunctival bevacizumab injection on corneal allograft survival after subsequent high-risk penetrating keratoplasty. Kaplan–Meier curves depicting the estimated probabilities of rejection-free corneal graft survival after penetrating keratoplasty. Estimated probabilities of rejection-free corneal graft survival were 92.9% after 1 year (number at risk: 23), 78.4% after 2 years (number at risk: 9), and 78.4% after 3 years (number at risk: 3) for all study eyes (non-HLA-matched and HLA-matched keratoplasties) (**A**) and 95.8% after 1 year (number at risk: 18), 75.8% after 2 years (number at risk: 6), and 75.8 after 3 years (number at risk: 2) for eyes only with non-HLA-7 matched keratoplasties (**B**). Mean follow-up time: 560 days; range: 59–1095 days. Vertical dashes indicate censored observations. CI, confidence interval. From Hos et al. 2019 [47].

As reviewed above, FND was shown to be a successful antiangiogenic as well as anti-lymphangiogenic [25] treatment strategy in our experimental study for preconditioning of pre-vascularized high-risk eyes prior to corneal transplantation and to promote subsequent graft survival [25]. However, clinical studies to investigate the efficacy of this preoperative approach were still missing. Very recently, we conducted a clinical pilot study that indicated that pre-transplant FND in combination with local anti-VEGF (bevacizumab) application seems to be effective in decreasing the incidence of allograft rejection after high-risk penetrating keratoplasty [47]. This interventional uncontrolled clinical study included 31 eyes of 31 patients with at least one corneal quadrant of corneal neovascularization [47] (Figure 11). FND was applied with subconjunctival injection of bevacizumab in all eyes before or during high-risk penetrating keratoplasty. FND and bevacizumab applications were repeated when visible reperfusion of pre-occluded vessels occurred on the day of penetrating keratoplasty [47], which was consistent with the largest report of FND in treating corneal neovascularization [137]. The initial results during follow-up (mean: 560 days; range: 59–1095 days) presented rejection-free allograft survival rates of above 90% after 1 year follow-up and about 80% after 2 and 3 years follow-up [47] (Figure 12) with no observation of complications. Additionally, this approach was safe and well-tolerated in all treated patients [47]. In comparison with the so far largest cohort study, reported by Williams and colleagues, which analyzed graft survival rates in vascularized versus nonvascularized recipients [138], the results of our study indicated an increase of rejection-free graft survival rates during the observation period, though the follow-up duration of our study is relatively short and longer follow-up in the included patients are not yet available. The limitations of this study are the relatively small number of included patients, the absence of internal controls as mentioned, and the heterogeneity of the patients in the aspect of vascularized quadrants as well as HLA-matching. Therefore, we need further controlled clinical trials with a larger number of patients and longer follow-up to verify the outcome of this pilot study. In addition, as discussed above [59], prospective investigations would be beneficial to examine whether, for instance, additional postoperative anti-angiogenic therapy might further improve corneal allograft survival. Recently, the combination of FND with VEGF Trap(R1R2) was shown to be effective in preventing FND-caused rebound vascularization and inflammation, and resulted in improved efficacy in terms of lymphatic and blood vessel regression [92]. Nonetheless, our initial results of this clinical pilot study suggest that pre-transplant (lymph)angioregressive preconditioning of recipient beds via FND in combination with subconjunctival application of anti-VEGF (bevacizumab) seems to induce allograft survival rates approximately similar to survival rates observed in non-vascularized corneal transplant recipients. 

The findings of these pilot studies of CXL and FND warrant further larger scaled cohort clinical trials with longer follow-up, which may help to translate these approaches into clinical practice.

## 7. Conclusions and Further Perspectives

Lymph-angiogenesis, lymphatic vessels, and lymphatic endothelial immune cell interactions critically contribute to corneal graft rejection via the mediating of undesired immune response after corneal transplantation in high-risk eyes. Modulation of lymphatic trafficking has been shown experimentally to be a novel and valid concept to promote corneal transplant survival. Blockade of immune cell trafficking to regional lymph nodes after transplantation was primarily achieved by inhibiting progressing lymph-angiogenesis or by regressing already existing, mature pathological lymphatic vessels. In terms of regressing mature lymphatic vessels, CXL or FND are potent strategies in the experimental setting prior to transplantation (“(lymph)angioregressive preconditioning of the host”) and thereby to significantly improve transplant survival in high-risk corneal transplantation. Although further large-scale prospective clinical trials are still needed, early positive evidence of clinical pilot studies using preoperative CXL or FND seems to confirm the novel therapeutic concept of improving corneal transplant survival by temporary preoperative lymphangioregresssion (and thereby inhibiting lymphatic immune cell trafficking). Additionally, in regards to lymphatic regression as well as graft tolerance, to apply CXL and/or FND in combination with anti-VEGF agents, e.g., bevacizumab or aflibercept, may offer more benefits. In fact, we recently demonstrated that the simultaneous application of anti-VEGFs prevents the FND-induced rebound neovascularization caused by surgical trauma-induced upregulation of VEGFs [92]. In clinical routine, the combination of FND with off-label bevacizumab is most widely used [28]. This concept of modulation of lymphatic trafficking may not only benefit patients with corneal transplants in the eye, but potentially may also be applicable to other transplant sites, such as heart, kidney, and lung transplantation. Whereas the precise role of lymphatics immediately postoperatively in diverse forms of transplantation is still unknown, again early evidence suggests a key role of lymphangiogenesis for the mediation of immune responses [2].
